# Comprehensive transcriptome profiling of Taiwanese colorectal cancer implicates an ethnic basis for pathogenesis

**DOI:** 10.1038/s41598-020-61273-y

**Published:** 2020-03-11

**Authors:** Shao-Min Wu, Wen-Sy Tsai, Sum-Fu Chiang, Yi-Hsuan Lai, Chung-Pei Ma, Jian-Hua Wang, Jiarong Lin, Pei-Shan Lu, Chia-Yu Yang, Bertrand Chin-Ming Tan, Hsuan Liu

**Affiliations:** 1 Graduate Institute of Biomedical Sciences, College of Medicine, Chang Gung University, Taoyuan, Taiwan; 2 Division of Colon and Rectal Surgery, Lin-Kou Medical Center, Chang Gung Memorial Hospital, Taoyuan, Taiwan; 3 Graduate Institute of Clinical Medical Sciences, College of Medicine, Chang Gung University, Taoyuan, Taiwan; 4 Department of Biomedical Sciences, College of Medicine, Chang Gung University, Taoyuan, Taiwan; 5 Molecular Medicine Research Center, Chang Gung University, Taoyuan, Taiwan; 6 Department of Microbiology and Immunology, College of Medicine, Chang Gung University, Taoyuan, Taiwan; 7 Department of Otolaryngology-Head & Neck Surgery, Chang Gung Memorial Hospital, Linkou, Taoyuan Taiwan; 8 Department of Neurosurgery, Linkou Medical Center, Chang Gung Memorial Hospital, Linkou, Taiwan; 9 Research Center for Emerging Viral Infections, Chang Gung University, Taoyuan, Taiwan; 10 Department of Cell and Molecular Biology, College of Medicine, Chang Gung University, Taoyuan, Taiwan

**Keywords:** Cancer genomics, Colorectal cancer, Biomarkers

## Abstract

Colorectal cancer (CRC) is one of the most commonly diagnosed cancers worldwide. While both genetic and environmental factors have been linked to the incidence and mortality associated with CRC, an ethnic aspect of its etiology has also emerged. Since previous large-scale cancer genomics studies are mostly based on samples of European ancestry, the patterns of clinical events and associated mechanisms in other minority ethnic patients suffering from CRC are largely unexplored. We collected 104 paired and adjacent normal tissue and CRC tumor samples from Taiwanese patients and employed an integrated approach – paired expression profiles of mRNAs and microRNAs (miRNAs) combined with transcriptome-wide network analyses – to catalog the molecular signatures of this regional cohort. On the basis of this dataset, which is the largest ever reported for this type of systems analysis, we made the following key discoveries: (1) In comparison to the The Cancer Genome Atlas (TCGA) data, the Taiwanese CRC tumors show similar perturbations in expressed genes but a distinct enrichment in metastasis-associated pathways. (2) Recurrent as well as novel CRC-associated gene fusions were identified based on the sequencing data. (3) Cancer subtype classification using existing tools reveals a comparable distribution of tumor subtypes between Taiwanese cohort and TCGA datasets; however, this similarity in molecular attributes did not translate into the predicted subtype-related clinical outcomes (i.e., death event). (4) To further elucidate the molecular basis of CRC prognosis, we developed a new stratification strategy based on miRNA–mRNA-associated subtyping (MMAS) and consequently showed that repressed WNT signaling activity is associated with poor prognosis in Taiwanese CRC. In summary, our findings of distinct, hitherto unreported biosignatures underscore the heterogeneity of CRC tumorigenesis, support our hypothesis of an ethnic basis of disease, and provide prospects for translational medicine.

## Introduction

Colorectal cancer (CRC) is one of the most prevalent types of malignancies worldwide^1^. Despite surgical resection and advances in radiotherapy and chemotherapy, it remains the third leading cause of cancer mortality globally^2^. The progression of CRC follows the adenoma–carcinoma sequence, developing from normal mucosa into adenoma and eventually into malignant adenocarcinoma^3^. The etiology of this malignancy can be classified as either hereditary, which represents about 10%–15% of the overall incidence and is attributable to mutation in *APC* or DNA mismatch repair (MMR) genes, or a more prevalent sporadic type, which is characterized by chromosomal instability (CIN), microsatellite instability (MSI), or CpG island methylator phenotype pathways^4,5^. Interestingly, several other risk factors of CRC have been documented, such as alcohol intake, obesity, smoking, sedentary lifestyle, a diet low in fruit and vegetables, and consumption of red meat^6,7^, suggesting an environmental and possibly an ethnic basis of pathogenesis.

MicroRNAs (miRNAs) are a class of small, noncoding single-stranded RNAs that are initially transcribed by RNA polymerase II and then subject to an elaborate maturation process. miRNAs are known to exert posttranscriptional gene silencing via sequence complementary targeting to the 3’ UTR of target mRNA, with the aid of the RISC complex^8–10^. Several miRNAs are reportedly associated with CRC progression. These include the tumor-suppressive let-7 that targets the *KRAS* transcript^11^; the *APC*-targeting miR-135 that triggers activation of WNT signaling and chromosomal instability^12^; the microsatellite instability-promoting miR-155 that downregulates the multiple mismatch repair genes *MLH1*, *MSH2*, and *MSH6*^13^; and the DNA methyltransferase-inhibiting miR-29, miR-143, and miR-342, which collectively alter overall DNA methylome profiles and consequently contribute to misexpression of key tumor suppressors and oncogenes^14–16^. However, given that malignant transformation typically involves molecular perturbations at the system level, biological relevance of miRNAs is best understood in the context of miRNA–mRNA regulatory networks. To this end, paired expression profiles of miRNAs and mRNA in tumor specimens are desired to enable the construction and interrogation of regulatory networks implicated in tumorigenesis.

Genome instability is considered a cancer hallmark that causes genome rearrangements, such as inversion, translocation, deletion, and duplication. When these genomic alterations take place between two genes, it may result in the generation of fusion genes that potentially produce chimeric proteins^17,18^. Some fusion partner genes harbor a tyrosine kinase domain and exhibit a consecutive kinase activity, and this may constitute an oncogenic property that further drives cancer progression (e.g., *ALK*, *NTRK*, *EGFR*, and *RET*)^19,20^. Owing to their intrinsic kinase activity, these fusion genes can also be turned into a drug-actionable target using kinase inhibitor^19,21–24^. Several prevalent fusion genes in CRC, such as *ALK*, *RET*, *ROS1*, *RSPO3*, *TCF7L1*, and *TCF7L2*, have been previously described to impact CRC progression^25–30^. The number of fusion genes observed in CRC is comparatively lower than in other cancers^19^. However, these fusion genes may play a large role in cancer progression, thus holding great potential as a therapeutic target. Hence, further investigation in the prevalence, relevance, and characteristics of fusion genes in CRC is necessary.

To systematically categorize tumor stage and progression at the molecular level, several proposed cancer subtyping systems have centered on mutational or transcriptomic profiles^31,32^. Given that cancer is a disease of genome instability, cancer mutation signatures represent an effective means to stratify patient samples. Such stratification has been known to impact the expectancy of survival^33–35^. In addition to mutation signatures, distinct transcriptome alterations associated with tumorigenesis also constitute an informative reporter of clinical and biological characteristics, warranting the development of transcriptome-based subtyping systems^36,37^. An indicator of the emerging prevalence and reliability of transcriptome-based subtyping systems is the increasing number of studies that have established and applied transcriptome-based subtyping systems to molecularly stratify and understand the clinical outcome of CRC^38–41^. Among these schemes, the Colorectal Cancer Subtyping Consortium (CRCSC) utilized data from six CRC studies and developed a robust classification system with four consensus molecular subtypes (CMSs)^36^. Each of the four subtypes is characterized by distinct expression profiles of oncogenic/tumor suppressive genes and/or pathways, mutation states of particular genes, and MSI. Importantly, these subtypes also correlate with patient survival – CMS1 exhibits poor survival after relapse, whereas CMS4 shows dismal prognosis – strengthening the translational potential of this system in prognosticating CRC. Interestingly, however, CMS subtyping of a Japanese cohort revealed a worse survival outcome in CMS1^42^, which is a different result from previous observations and thus reinforces the ethnic or regional basis of pathogenesis.

Both genetic and environmental factors have been attributed to the pathogenesis of colorectal cancer (CRC). Given the recent upsurge in CRC incidence in different countries, an ethnic aspect of its etiology has emerged but remains largely unexplored. To address this issue, we collected 104 paired and adjacent normal tissue and CRC tumor samples from Taiwanese patients and deciphered their transcriptomic signatures. Comprehensive transcriptome sequencing analysis was combined with the analyses of profiles of RNAs, microRNAs (miRNAs), miRNA–mRNA network, fusion genes, and cancer subtypes. The comparison of transcriptomic profiles between The Cancer Genome Atlas (TCGA) and Taiwanese CRC samples reveals a moderate extent of correlated expression. We also identified novel CRC-associated fusion genes. Intriguingly, in comparison with the TCGA dataset, the Taiwanese CRC patient cohort exhibited a similar distribution of subtype assignment but showed distinct clinical outcomes among subtypes. To further investigate the miRNA–mRNA regulation network underlying the poor outcome, we constructed patient groupings according to miRNA–mRNA-associated subtyping (MMAS), which was previously applied in other cancer types^43–45^, We then revealed based on the identified regulons that subtype-specific poor outcome is associated with repressed WNT signaling. Viewed together, our study identified several molecular and clinical distinctions in the Taiwanese CRC samples compared with the samples from Western countries, providing mechanistic insights into the nature of the disease and a strong support for an ethnic basis in CRC tumorigenesis. In summary, our findings of distinct, hitherto unreported biosignatures support our hypothesis of an ethnic basis of CRC tumorigenesis and further provide mechanistic insights into the nature of the disease and translational medicine potential of the new system.

## Results

### Comprehensive profiling of Taiwanese CRC transcriptomes reveals cohort-specific alterations

To comprehensively catalog the dysregulated transcriptomic alterations underlying ethnically or regionally specific CRC, we first recruited ethnic Taiwanese patients admitted to the Chang Gung Memorial Hospital. The clinical characteristics and demographics of our cohort are outlined in Table 1, which were further analyzed for their association with the overall survival (OS) and disease-free survival (DFS) of the patients (Table 2). Univariate analysis then revealed that TNM staging (P value = 0.0362 and 0.0341 for DFS and OS, respectively) and metastasis lymph node number (P value = 0.00652 and 0.0114 for DFS and OS, respectively) were significantly associated with DFS and OS and that such histological grades could be a risk factor for OS. Next, matched pairs of CRC tumors and adjacent normal tissues from the same patients (n = 104) were collected and subjected to both RNA-seq and small RNA-seq analyses. Detailed statistics of sequencing data are summarized in Figure S1A–B, Additional file 1. We generated 36.3 ± 10.5 million and 22.2 ± 10.7 million mean mapped read counts for RNA-seq and small RNA-seq, respectively. To control for the quality of our deep sequencing results, we first called variants from all samples and analyzed them by BAM-matcher, which examines sample pairing on the basis of the fraction of common SNPs among variants^46^ (Additional file 1: Figure S1C). The results subsequently showed that the samples were properly paired and suitable for further analysis.

**Table 1:** Clinical characteristics of CRC patients in this study.

Characteristics	CRC Patients (N = 104)
**Gender**	
F	45 (43)
M	59 (57)
**TNM stage**	
I	18 (17)
II	29 (28)
III	42 (40)
IV	13 (12)
TisN0	2 (2)
**Location**	
Left colon	42 (40)
Rectum	41 (39)
Right colon	12 (12)
Transverse colon	9 (9)
**Age**	
min	39
median (IQR)	62.00 (55.75, 68.25)
max	85
mean (sd)	61.86 ± 10.40
**Alcohol**	
No	67 (64)
Yes	37 (36)
**CEA_conc**	
min	0.5
median (IQR)	2.70 (1.20, 6.08)
max	1957
mean (sd)	31.97 ± 195.50
**Family_ca_HX**	
Hnpcc	3 (3)
No	59 (57)
Unk	1 (1)
Yes	41 (39)
**Histological grade**	
Well differentiated	91 (88)
Poor differentiated	13 (12)
**Smoke**	
No	60 (58)
Yes	44 (42)

Family ca HX, Family cancer history; Hereditary Nonpolyposis Colorectal Cancer, HNPCC; Unk, Unknown.

**Table 2:** Univariate analysis of disease free survival (DFS) or overall survival (OS).

Characteristics	Hazards ratio (DFS)* (95% confidence interval) (N = 91)	P value (DFS)	Hazards ratio (OS) (95% confidence interval) (N = 104)	P value (OS)
**Age**				
	0.9983 (0.95–1.05)	0.947	0.9691 (0.919–1.02)	0.248
**Gender**				
Female	1.000 (Reference)	0.311	1.000 (Reference)	0.236
Male	1.84 (0.566–5.98)		2.231 (0.592–8.41)	
**Overall pathological stage (TNM)**				
TisN0** + I–II	1.000 (Reference)	0.0362	1.000 (Reference)	0.0341
III–IV	3.971 (1.09–14.4)		9.235 (1.18–72.2)	
**Tumor size**				
<2.5 cm	1.000 (Reference)	0.48	1.000 (Reference)	0.217
2.5 cm – 5 cm	3.316 (0.424–25.9)		1.373 (0.16–11.8)	
> 5 cm	2.215 (0.201–24.4)		3.707 (0.432–31.8)	
**Metastasis lymph node number**				
<2	1.000 (Reference)	0.00652	1.000 (Reference)	0.0114
≥ 2	5.999 (1.65–21.8)		14.23 (1.82–111)	
**Smoke**				
No	1.000 (Reference)	0.118	1.000 (Reference)	0.13
Yes	2.445 (0.798–7.49)		2.587 (0.756–8.85)	
**Alcohol**				
No	1.000 (Reference)	0.325	1.000 (Reference)	0.433
Yes	1.73 (0.581–5.16)		1.607 (0.49–5.27)	
**CEA (**>**5** **ng/ml)**				
No	1.000 (Reference)	0.08	1.000 (Reference)	0.255
Yes	2.657 (0.89–7.93)		1.993 (0.608–6.53)	
**Histological grade**				
Well differentiated	1.000 (Reference)	0.349	1.000 (Reference)	0.000103
Poor differentiated	2.055 (0.455–9.28)		10.62 (3.22–35)	
**Splenic flexure**				
Right	1.000 (Reference)	0.241	1.000 (Reference)	0.783
Left	3.393 (0.441–26.1)		1.24 (0.268–5.74)	

*Stage 4 patients were excluded in DFS analysis; **adenoma (T vs. N0).

**Figure 1:**
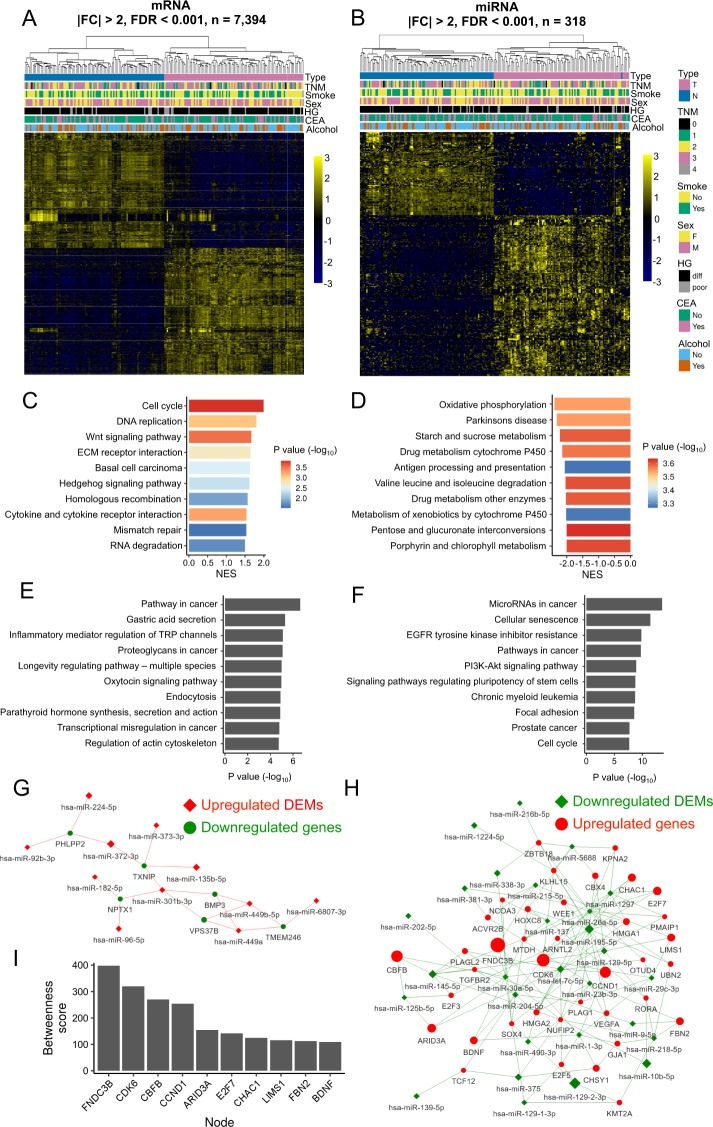
Comprehensive profiling of Taiwanese CRC transcriptomes reveals cohort-specific alterations. Hierarchical clustering of (**A**) DEGs and (**B**) DEMs from Taiwanese CRC patients demonstrating a disturbed expression profile between normal (blue) and tumor (red) tissues. GSEA charts of top ten significantly enriched (P value < 0.05) upregulated (C, NES > 0) and downregulated (D, NES < 0) KEGG pathways, where each row represents indicated NES value and the color shows the significance of enrichment result in -log_10_ transformed P value. Detailed enrichment results are listed in the Table S3, Additional file 2. Gene set over-representation analysis of top ten (**E**) upregulated and (**F**) downregulated DEMs targeting mRNAs enriched pathways. Construction of miRNA–mRNA correlation network based on (**G**) upregulated DEMs/downregulated DEGs and (**H**) downregulated DEMs/upregulated DEGs, in which DEGs are targeted by more than two miRNAs. miRNAs, squares; mRNA, circles; upregulated, red; downregulated, green. (**I**) Bar plot of betweenness scores for top ten nodes from networks (**G**) and (**H**).

Next, principal component analyses (PCA) of the RNA-seq and small RNA-seq data were performed to comparatively characterize the overall transcriptome profiles. To this end, the PCA plots reveal distinct expression profiles corresponding to the disease states (Additional file 1: Figure S1D–E). Genes exhibiting CRC-associated differential expression patterns were identified using DESeq. 2, which yielded 7,394 (3,884 upregulated and 3,510 downregulated) mRNAs and 318 (210 upregulated and 108 downregulated) miRNAs differentially represented in the CRC tumor vs. normal tissues (|fold change | >2, FDR < 0.001, Additional file 2: Tables S1–2). The overall distributions of these transcriptome changes in relation to various clinical attributes were further characterized by hierarchical clustering and shown by a heatmap (Fig. 1A,B). Given that tumorigenic progression is typically associated with alterations in molecular pathways^47,48^, we next sought to explore dysregulated pathways in Taiwanese CRC based on our RNA-seq data. Pathway analysis of the upregulated genes using Gene Set Enrichment analysis (GSEA) reveals significant enrichment in several pathways, such as the cell cycle, DNA replication, and WNT signaling pathway (Fig. 1C, Additional file 2: Table S3). Conversely, downregulated genes were enriched in factors associated with oxidative phosphorylation, Parkinson’s disease, and starch and sucrose metabolism pathways (Fig. 1D).

Concurrent profiling of expressed mRNAs and miRNAs constitutes a strong basis for the in-depth assessment of the regulatory relationships between miRNAs and mRNAs in Taiwanese CRC transcriptomes. Toward this end, we retrieved miRNA–target interactions (MTIs) from miRTarBase, which archives experimentally validated miRNA–target interaction networks^49^; we also retrieved computational MTIs from TargetScan and integrated both experimental and computational data to generate a reliable miRNA–target prediction ref. ^50^. By cross-referencing MTIs with DEMs and the targeted genes identified in our cohort (|fold change | >2, FDR < 0.001; *r* < 0, P value <0.05), a global representation of miRNA–target interaction networks in Taiwanese CRC was constructed, in which 67 miRNAs and 1,529 mRNAs interconnected to form 2,166 unique miRNA–mRNA regulatory pairs. To further understand the potential outcome of these coordinated transcriptome alterations, we performed gene set over-representation analysis on miRNA-targeted mRNAs in these regulatory hierarchies and further discovered that mRNAs targeted by upregulated DEMs in these MTIs are enriched in genes involved in the pathways of cancer, gastric acid secretion, and inflammatory mediator regulation of TRP channels (Fig. 1E, Additional file 2: Table S4). Conversely, mRNAs targeted by downregulated DEMs were enriched in the components of cancer microRNAs, cellular senescence, and EGFR tyrosine kinase inhibitor resistance (Fig. 1F, Additional file 2: Table S4).

Our miRNA–mRNA networks illustrate thousands of nodes and edges representing complex interactions. However, critical nodes and edges within the networks remained largely elusive. It has been reported that genes with higher degrees of connections within a network may potentially play important biological roles^51,52^. Therefore, to identify the critical subnetworks underlying CRC progression, we next focused on genes targeted by more than two miRNAs. We first selected network nodes that were either DEGs or DEMs, based on the dysregulated miRNAs revealed by differential expression analysis (Fig. 1A,B). These nodes were constructed into miRNA–mRNA subnetworks, with each node size being assigned a betweenness centrality score (Fig. 1G–I), which weighs the cluster connectivity and potential impact of a node^52^. Interestingly, several drug actionable genes were identified in this subnetwork, such as CKD6, CCND1 (PALBOCICLIB and Ribociclib), and VEGFA (AFLIBERCEPT)^53^. Taken together, these observations illustrate the extensive and distinct fluctuations in the miRNA–mRNA regulatory cascades associated with Taiwanese CRC, with potential impact on the expression and outcome of certain tumorigenesis-associated molecular pathways.

### Comparison between Taiwanese CRC data and The Cancer Genome Atlas (TCGA) reveals regional differences

**Figure 2:**
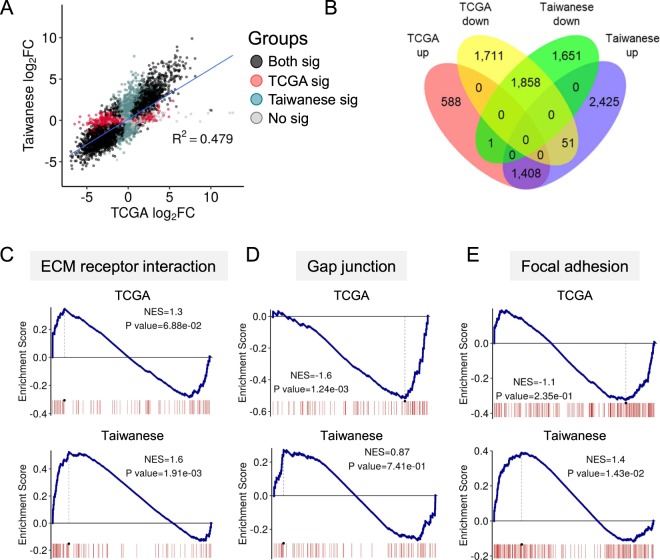
Comparison between Taiwanese and The Cancer Genome Atlas (TCGA) CRC dataset. Comparison expression profiles between TCGA and Taiwanese CRC dataset are demonstrated by (**A**) a scatterplot of gene tumor/normal fold changes (FC) with log_2_ transformed fold changes (FC) (*R*^2^ = 0.478, P value <0.001). Genes are colored in red, green, and black by the status of significant expression in TCGA, Taiwanese CRC, and both datasets, respectively. Sig: significantly expressed gene (FDR < 0.001). (**B**) Venn diagram shows the overlapping of upregulated/downregulated DEGs between TCGA and Taiwanese dataset. (C-E) Demonstration of distinct pathway expression patterns between TCGA and Taiwanese CRC dataset using GSEA.

We next compared the overall transcriptomic profiles of our Taiwanese cohort with the TCGA dataset, which comprises data from patients primarily of European ancestry. For this purpose, RNA-seq data for the 50 pairs of normal tissue and matched tumors of colon adenocarcinoma and rectum adenocarcinoma were downloaded from TCGA for transcriptome profiling using the same pipeline. Differential expression analysis first uncovered 5,617 DEGs, of which 1,997 and 3,620 genes were upregulated and downregulated, respectively (Additional file 2: Table S5). While there was a moderate extent of expression correlation between the two datasets, as shown by the scatter plot of Fig. 2A, genes that were significantly expressed (FDR < 0.001) exhibited markedly different patterns between the TCGA and Taiwanese data (*R*^2^ = 0.479, Fig. 2A). Further comparison at the gene level shows that a sizable fraction of the DEGs was uniquely detected in either dataset (Fig. 2B), indicating prominent levels of distinction at the gene level. Interestingly, GSEA analysis demonstrated a high level of similarity in the overall pathway enrichment between the two CRC datasets (Additional file 1: Figure S2A–B, Additional file 2: Table S3 and S6). In particular, components of the tumor- or CRC-associated molecular pathways^54^, such as cell cycle, DNA replication, mismatch repair, starch and sucrose metabolism, drug metabolism, and cytochrome p450 pathways, were dysregulated in both datasets (Additional file 2: Table S3 and S6). Nonetheless, we noticed differential representation of pathways between cohorts, such as those related to ECM receptor interaction, gap junction, and focal adhesion; these pathways were comparatively upregulated in the Taiwanese dataset (Fig. 2C–D, Additional file 1: Figure S2C–E). Comparison of the disturbed genes and pathways of the TCGA and Taiwanese datasets showed a certain degree of similarity, suggesting that the overall progression of CRC could be similar for both groups. However, metastasis-associated pathways, ECM receptor interaction, gap junction, and focal adhesion showed distinct patterns between the two datasets^55–57^. Furthermore, the proportion of stage 4 Taiwanese CRC patients is lower than that reflected in TCGA (16% and 12.5% of stage 4 patients for TCGA and Taiwanese datasets, respectively.). This suggests a possibility of distinct development of metastasis between the cohorts represented by these two datasets.

### Novel gene fusion events in Taiwanese CRC

Tumor cells are prone to gene fusion events owing to their intrinsically unstable genomes. Mounting evidence indicates that these fusion genes are associated with oncogenic properties and are thus therapeutically actionable targets^17,20^. Therefore, we next identified the fusion genes in Taiwanese CRC by analyzing the RNA-seq data using the STAR-Fusion tool^58^. To this end, we filtered candidate fusion genes by the following criteria: junction read counts (greater than five), in-frame fusion, and absence in the matched normal tissue. A total of 22 fusion genes were identified in 16 of the patient samples (Additional file 2: Table S7). Given that clinical attributes, such as MSI, are potentially correlated with the incidence of fusion genes^59^, we next examined this possibility. However, we did not identify any preponderance of clinical attributes (Table 3) among these fusion-positive individuals.

**Table 3:** Association between fusion genes and clinical attributes.

	No.	Presents of fusion genes		P value
		No	Yes	
**Sex**				
Female	45	40 (88.9)	5 (11.1)	0.412
Male	59	48 (81.4)	11 (18.6)	
**TNM stage**				
I	18	14 (77.8)	4 (22.2)	0.93
II	29	25 (86.2)	4 (13.8)	
III	42	36 (85.7)	6 (14.3)	
IV	13	11 (84.6)	2 (15.4)	
TisN0	2	2 (100)	0 (0)	
**Location**				
Left colon	42	38 (90.5)	4 (9.5)	0.39
Rectum	41	34 (82.9)	7 (17.1)	
Right colon	12	9 (75)	3 (25)	
Transverse colon	9	7 (77.8)	2 (22.2)	
**Aloohol**				
No	67	57 (85.1)	10 (14.9)	1
Yes	37	31 (83.8)	6 (16.2)	
**CEA (**>**5** **ng/ml)**				
No	72	61 (84.7)	11 (15.3)	1
Yes	32	27 (84.4)	5 (15.6)	
**Family ca HX**				
HNPCC	3	2 (66.7)	1 (33.3)	0.239
No	59	53 (89.8)	6 (10.2)	
Unk	1	1 (100)	0 (0)	
Yes	41	32 (78)	9 (22)	
**Histological grade**				
Well differentiated	91	79 (86.8)	12 (13.2)	0.113
Poor differentiated	13	9 (69.2)	4 (30.8)	
**Smoke**				
No	60	51 (85)	9 (15)	1
Yes	44	37 (84.1)	7 (15.9)	

Family ca HX, Family cancer history; Hereditary Nonpolyposis Colorectal Cancer, HNPCC; Unk, Unknown.

**Figure 3:**
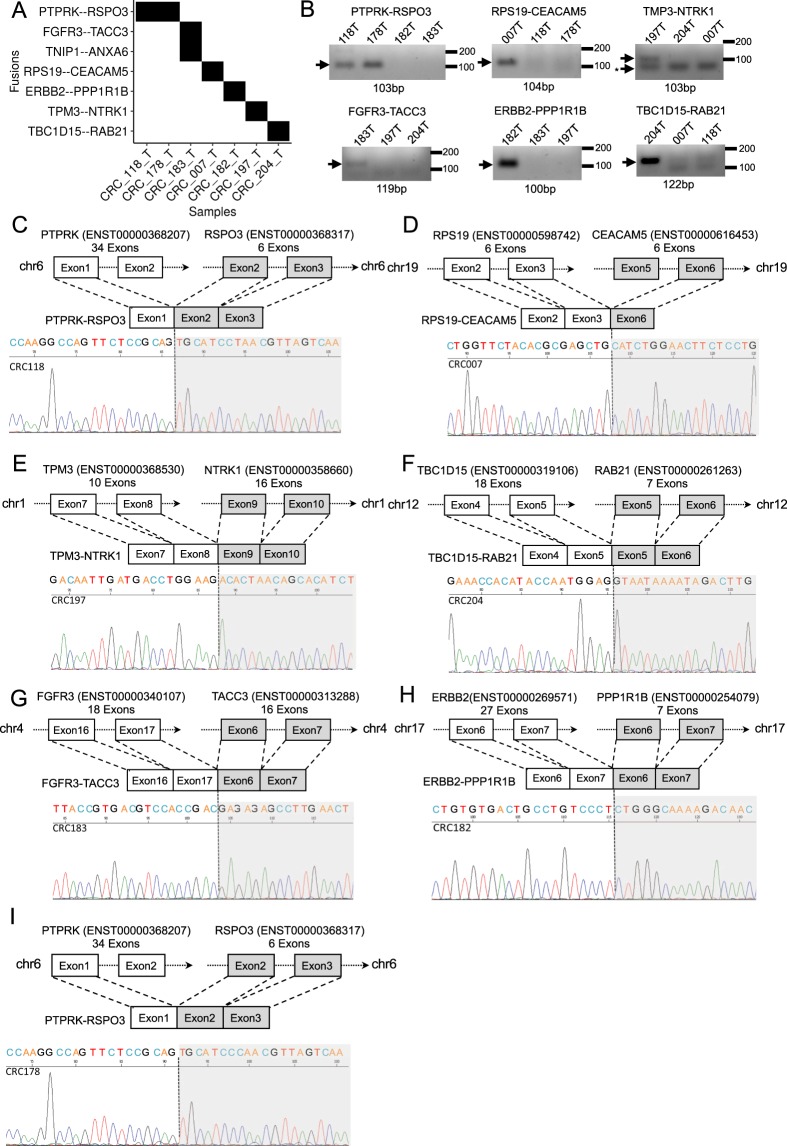
Landscape of expressed fusion genes in Taiwan CRC cohort. (**A**) Summarized chart of fusion genes and samples. The corresponding row and column of a given black square indicates a detection of fusion gene event in a sample. (**B**) RT-PCR validation of fusion genes. Arrows labeled with fusion gene name denote the expected PCR product, whereas asterisks correspond to non-specific signal. Schematic illustration shows the breakpoint localization (top) and sanger sequencing chromatogram (bottom) for (C and I) *PTPRK-RSPO3*, (**D**) *RPS19-CEACAM5*, (**E**) *TMP3-NTRK1*, (**F**) *TC1D15-RAB21*, (**G**) *FGFR3-TACC3*, and (**H**) *ERBB2-PPP1R1B* with corresponding samples.

We then focused on known gene fusion events, as their detection is essentially more reliable than those of novel fusion genes. By installing additional filters in our selection, we thus excluded events not annotated in the STAR-Fusion databases (Fig. 3A). In addition, we annotated fused genes encoding the kinase domain, owing to the importance of kinases-mediated signaling in tumorigenesis (Additional file 2: Table S7)^60^. As positive controls for this analysis, we analyzed two CRC-associated recurrent fusions, *PTPRK-RSPO3* and *TPM3-NTRK1*, in three of our patient samples (Fig. 3A)^27,29^. Interestingly, our cohort did not express other known CRC-associated fusion events, such as those involving *ALK*, *RET*, *ROS1*, and *TCF7L2*^25,28,30^, implying a cohort-specific genomic alteration. We also identified three fusion events, *RPS19-CEACAM5*, *TBC1D15-RAB21*, and *TNIP1-ANXA6*, in which one partner has been implicated in non-CRC tumorigenesis^61–63^. Interestingly, we identified two novel CRC fusions, *ERBB2-PPP1R1B* and *FGFR3-TACC3*, comprising genes encoding the kinase domain (Fig. 3A). To further validate these structurally anomalous transcripts, we conducted RT-PCR assays and Sanger sequencing for the fusion pairs *PTPRK-RSPO3*, *TMP2-NTRK*, *FGFR3-TACC3*, *RPS19-CEACAM5*, *TC1D15-RAB21*, and *ERBB2-PPP1R1B*, and successfully confirmed their expression in the corresponding samples (Fig. 3B–N).

### Transcriptome-based subtyping of Taiwanese CRC patients

During the progression of tumor growth, cellular heterogeneity arises as a result of diverse mutation signatures, expression profiles, and tumor malignancies, contributing to the differentiation of tumor subtypes among patients. Importantly, distinct subtypes of tumors are highly correlated with disease outcome and can potentially result in variable responses to therapies^37,38,42^. Therefore, relating tumor subtypes to clinical relevance can help illuminate disease mechanisms and develop precision medicine for CRC. To address this issue, CRCSC previously developed a robust molecular signature approach for subtyping CRC, resulting in the identification of four clinically relevant CMS for CRC^36^. Another approach, the CRCA, collected two datasets and used NMF to define a 786-gene classifier for assigning samples into five subtypes based on the cell types of the colon crypt^37^. The distributions of patients, molecular signatures, and survival outcome for each subtype were well documented by these studies. However, since the profiling data were obtained from patients of predominantly European ancestry, whether this classification scheme could be similarly applied to patients of other ethnicities remains unknown.

To test this possibility, we first applied the CRCSC and CRCA^36,37^ systems to assign subtypes among the Taiwanese CRC patients based on the nearest template prediction, resulting in 88.5% and 91.3% of patients being successfully assigned, respectively (Additional file 1: Figure S3A–B). These approaches have previously shown that CMS4 and stem-like subtypes express signatures of EMT and TGF-β signaling that correspond to worse OS or DFS survival^36,37^. We then examined whether Taiwanese CRC patients exhibit similar subtype-specific outcomes by performing OS and DFS survival and Cox proportional hazards analyses on the CRCSC and CRCA assigned patients (Additional file 1: Figure S3C–F). However, the stratified survival analysis did not reveal analogous patterns of patient survival in our cohort; in fact, no significant difference in survival outcome was observed among subtypes. We further compared the overall distribution of CRCSC and CRCA assigned subtypes between Taiwanese and TCGA patients. Our results indicate that while the assigned distributions by CRCSC and CRCA are similar between the two datasets (*SD* = 3.5% and 2.9%, respectively; Additional file 2: Table S8), the prognosis of each subtype was largely different between the two datasets (*SD* = 13% and 10.3%, respectively; Additional file 2: Table S8). For instance, whereas the CMS4 and stem-like subtypes in the TCGA dataset are prone to death, such is not the case in the Taiwanese dataset. This observation implies that cohort-specific, distinct molecular pathways are associated with the subtype-specific outcomes. Nevertheless, it remains formally possible that other factors such as distributions of CRC stages might also contribute to the inter-cohort differences in subtype-associated disease outcome.

**Figure 4:**
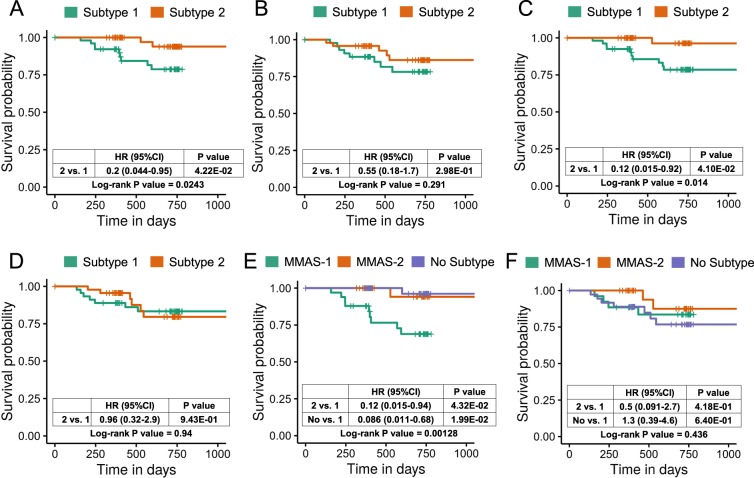
Identification of miRNA–RNA-associated subtypes using unsupervised NMF clustering. Kaplan–Meier plot for overall survival and disease-free survival analysis. (**A**) OS and (**B**) DFS analysis for Taiwanese CRC patients stratified by groups of mRNA NMF clustering. (**C**) OS and (**D**) DFS analysis for Taiwanese CRC patients stratified by groups of miRNA NMF clustering. (**E**) OS and (**F**) DFS analysis for Taiwanese CRC patients stratified by concordant assignment of mRNA and miRNA NMF clustering.

Because CRCSC and CRCA classification approaches did not show consistent subtype-specific outcomes for the Taiwanese CRC patients (Additional file 1: Figure S3C–F), we next devised an independent classification scheme based on our multidimensional transcriptome data, aiming toward a robust and biologically relevant discrimination in terms of miRNA–mRNA regulation. To this end, we used NMF to classify tumor samples on the basis of mRNA and miRNA expression profiles and screened for subtypes differing in terms of OS or DFS (see Methods) and concordant sample grouping in both RNA molecule data. The most optimal NMF classification results are based on a 400-mRNA panel and a 50-miRNA panel, each of which clustered samples into two distinct groups, subtypes 1 and 2 (Additional file 1: Figure S4A–B, Additional file 2: Tables S9, 10). Moreover, both of the mRNA and miRNA subtype classifiers show that subtype 1 is linked to worse OS outcome (Fig. 4A,C, mRNA and miRNA log-rank test P value = 0.0243 and 0.014, respectively), but not in DFS outcome (log-rank test P value = 0.291 and 0.94, respectively; Fig. 4B,D). Total of 33 and 32 patients were concordantly assigned by both datasets to subtypes 1 and 2, respectively, as shown by a contingency table of sample groupings from the NMF classification (Table 4). Fisher’s exact test (P value = 0.01815) further indicates a significant extent of correlation between the mRNA- and miRNA-based groupings. Based on this concordance, we regrouped the patients according to the new “miRNA–mRNA-associated subtypes” (or MMAS) 1 and 2, whereas the remaining patients with ambivalent classification were defined as non-MMAS. A stratified survival analysis on the new MMASs again showed the worst OS outcome in MMAS-1 (but not DFS) among the three groups (Fig. 4E–F). The Cox proportional hazards analysis also shows that MMAS-1 exhibits considerably poorer outcome than MMAS-2 (Fig. 4E). In summary, these results have uncovered clinically relevant transcriptome signatures that represent key determinants underlying survival outcome of CRC patients, further supporting the notion that miRNA–mRNA molecular interactions contribute to the survival outcome of CRC patients.

**Table 4:** Contingency table of samples groupings from NMF classification using miRNA and mRNA data.

		miRNA	
		1	2
mRNA	1	33	19
	2	20	32

### Characterization of NMF-assigned subtypes shows differential representation of signatures

**Figure 5:**
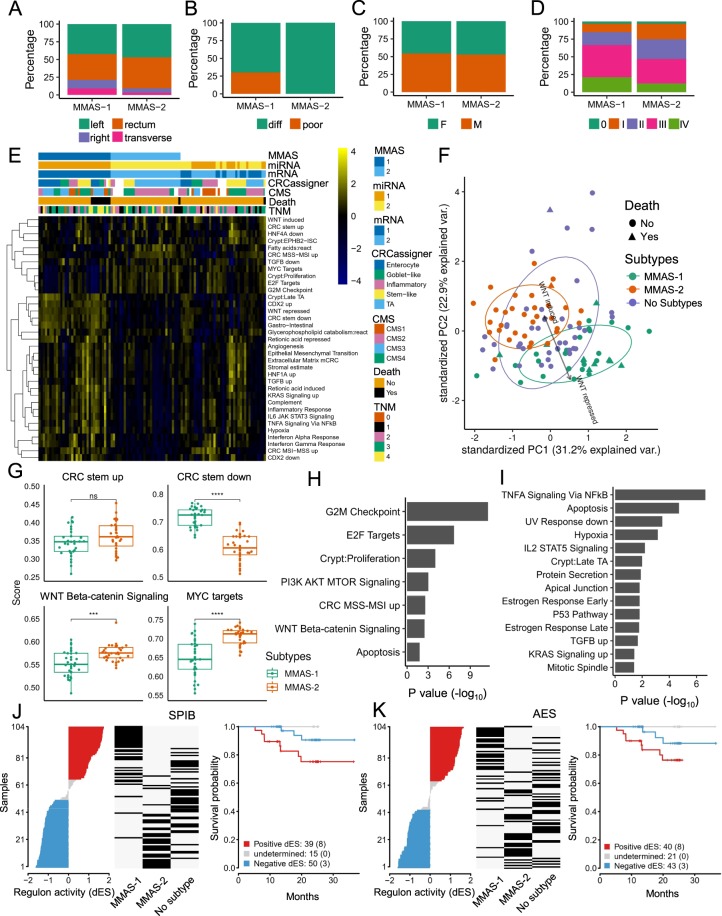
Characterization of NMF-assigned subtypes shows differential representation of signatures. Distribution of (**A**) tumor localization, (**B**) histological grade, (**C**) sex, and (**D**) TMN staging across MMAS assigned samples. Association between attributes and samples are examined using Fisher’s exact test, in which the P value are 6.42e-01 (**A**), 8.77e-04 (**B**), 1.00e + 00 (**C**), and 6.07e-01 (**D**), respectively. (**E**) Heatmap of ssGSEA result for 104 samples with top 35 most deviated pathways is shown. Columns are sorted by MMAS groups and death events. (**F**) PCA of ssGSEA result for 104 tumor tissues. Samples with top 20 most deviated pathways are plotted as function of PC1 and PC2. The black arrow indicates the direction of variable loadings for the corresponding pathways. (**G**) Box plot of pathway enrichment score for CRC stem up, CRC stem down, WNT/β-catenin signaling, and MYC target pathways. Comparison of enrichment score between MMASs is performed using the Mann–Whitney test. (**H**) & (**I**) Gene set over-representation analysis of genes targeted by DEMs (differentially expressed in the MMAS-1 vs. MMAS-2 comparison), and enriched pathways of the (**H**) upregulated and (**I**) downregulated DEMs-targeting network are shown. The TF regulon activity for (**J**) SPIB and (**K**) AES regulons was analyzed by RTN on the basis of (left panel) differential enrichment scores (dES), (middle panel) MMAS assignment status, and (right panel) Kaplan–Meier survival plot. Left panel: Differential enrichment scores (dES) for 104 CRC tissues, where red and blue indicate activated and repressed regulon activity, respectively. Middle panel: MMAS assignment status. Black bars indicate MMAS assignment status for each sample, which correspond to the left panel by position in row. Right panel: Kaplan–Meier survival plot of OS stratified by activated/repressed regulon status. The significance of the survival outcome difference is based on log-rank test. (ns, no significance; ***P value ≤ 0.001; ****P value ≤ 0.0001, Mann–Whitney test.).

To further characterize the differences between the subtypes, we examined the distribution of sample attributes among MMASs. We first discovered that histological grade was significantly associated with MMASs, but did not detect any association for tumor localization, sex, and TNM staging (Fig. 5A–D). Given the distinct clinical outcomes, we hypothesized that transcriptome profiles might also be altered between these MMASs. Therefore, we performed subtype-specific differential expression analysis, resulting in the identification of 5,387 DEGs and 331 DEMs (|fold change | >1.5, FDR < 0.05, Additional file 1: Figure S5A–B; Additional file 2: Table S11, 12). Next, we examined CRC-associated signatures among patients by using ssGSEA (Fig. 5E)^64^. The PCA reveals MMAS-1 and MMAS-2 clustered into two definitive groups, in contrast to the scattered pattern of the “non-MMAS subtype,” suggesting that MMASs exhibit distinct CRC-associated signatures (Fig. 5E–F). The PC1 and PC2 derived from variable loadings of PCA could generate vectors, of which the direction delineates signatures for the given samples. Interrogating the variable loadings of PCA identified attributes, such as late TA, CDX2 up, WNT-repressed, CRC stem down, and gastrointestinal pathways as the major variable pathways expressed in the MMAS-1 patients (Fig. 5F, Additional file 1: Figure S5C). In contrast, the MMAS-2 patients are enriched in WNT-induced and CRC stem-up pathways (Fig. 5F, Additional file 1: Figure S5C). In line with the potentially perturbed WNT signaling, our analysis further shows that WNT signaling and the MYC and CRC stem-down pathways were differentially expressed to a significant extent between MMASs (Fig. 5G). To examine the robustness of our MMAS approach, we repeated the above analyses with resampling of 80% samples and event sampling ratio of 5 (OS). We found that profiles of resampled data consistently corresponded to repressed WNT signaling pathways and poor outcome for OS in MMAS-1 (Additional file 1: Figure S6). Viewed together, these results reinforce the clinical and molecular distinctions between MMASs and further demonstrated that WNT signaling pathway is the predominant pathway discriminating between the MMASs.

While the MMAS classification is based on both mRNA and miRNA panels, it is unclear whether this tumor subtyping involves any coordinated miRNA–mRNA regulation. To explore this possibility, we performed pathway analysis of the miRNA-regulated gene networks (see Methods) and looked for any overlap with the enriched pathways of mRNA genes. We found a high correlation between enriched pathways from both transcriptome signatures, in which 5/7 and 13/14 of miRNA upregulated and downregulated pathways (MMAS-1 vs. MMAS-2; DEMs: |fold change | >1.5, FDR < 0.05; miRNA-mRNA: *r* < 0, P value < 0.05) corresponded to the downregulated and upregulated gene pathways, respectively (Fig. 5H–I, Additional file 2: Table S13). These results illustrate that the subtyping and outcome of the MMASs are governed not only by distinct expression profiles but also by the two-tier coordination of miRNA–mRNA regulation.

Interestingly, in line with the poor outcome of MMAS-1, the MMAS-1-associated transcriptome was enriched in several poor prognosis-associated pathways, such as WNT signaling, EMT, TGF-β, and stem cell pathways. Given that several TFs have been implicated in the gene expression associated with tumorigenesis and survival outcome^65–68^, we next assessed the contribution of TFs in the subtype/outcome-specific gene networks. To identify MMAS-associated risk TFs, we performed the reconstruction of transcriptional networks (RTN) analysis by examining TF regulon activity for each sample^69^. The result shows that the SPIB regulon has the highest association with MMAS (Fig. 5J; Fisher’s exact test P value = 3.65e-13), whereby the activated regulon is correlated with the poor outcome (log-rank test P value = 0.025) of MMAS-1. Furthermore, we uncovered activated AES regulon activity in association with MMAS-1 (Fig. 5K; Fisher’s exact test P value = 7.3e-08; log-rank test P value = 0.031). Notably, given that AES suppresses WNT signaling^70,71^, our analysis in this part strengthened the link of WNT signaling to the stratification and clinical outcome of MMASs. Finally, upon further examination, we noticed that some MMAS-1 patient samples exhibit upregulated CMS1 signature pathways (inflammatory response and interferon response) and CMS4 signature pathways (angiogenesis, EMT, extracellular matrix mCRC, and TGF-β up)^36^. This distinct combination of signatures strongly suggests that the MMAS-1 subtypes can express multiple pathways in addition to the suppressed WNT signaling. Taken together, this molecular characterization of MMASs underscores the heterogeneity of and regional predisposition to CRC tumorigenesis, thus serving as an important basis for precision medicine of this tumor type.

## Discussion

*ALK*, *ROS1*, and *NTRK* fusions are well-known CRC-associated fusions; they have been classified as a new subtype of metastatic CRC that exhibits poor prognosis^72^. It has also been reported that kinase inhibitor intervention can be applied for these fusions^28,73,74^. In addition to such targeted therapy, fusion genes are translated into peptides, which can potentially act as a neoantigen and can thus be treated with cancer immunotherapy^75,76^. These findings extend the treatment options of CRC patients to further increase their chances for survival. In the present study, we observed several fusion genes, and 7 out of 104 (6.7%) patients were validated with corresponding fusions. Of these, *PTPRK-RSPO3* and *TPM3-NTRK1* are CRC-associated recurrent fusions that are amenable to the porcupine inhibitor and kinase inhibitor interventions, respectively^73,77^. Notably, the *FGFR3-TACC3* fusions recurrent in urothelial bladder carcinoma, glioblastoma (GBM), head–neck squamous cell carcinoma (HNSC), and low-grade glioma show stable and minor response to FGFR inhibitor treatment^19,78^. For fusions such as *ERBB2-PPP1R1B*, *RPS19-CEACAM5*, *TBC1D15-RAB21*, and *TNIP1-ANXA6* without appropriate actionable targets for drug therapy, the neopeptides generated from these fusions can potentially be the target of immunotherapy. According to an National Comprehensive Cancer Network (NCCN) guideline, larotrectinib was recently added as a treatment for metastatic colorectal cancer patients positive for *NTRK* gene fusion^79,80^. Therefore, these fusion genes have a great potential to be a therapeutic target in a precision medicine approach.

Some MMAS-1 patients who died were enriched in the CMS4 signature reflecting the EMT and TGF-β pathways (Fig. 5E). It is intriguing that these patients had not been assigned as CMS4. From this observation, some considerations arise: first, we used NTP to perform the classification for the patients. NTP classifies samples based on the most expressed gene of a given subtype; therefore, it is possible that both CMS4 and CMS1/2/3 signatures are highly expressed, but the CMS1/2/3 signature genes are expressed at a higher level than CMS4, resulting in CMS1/2/3 classification. Previously, Ma *et al*. suggested that the CRC transcriptome exhibits a continuous profile among subtypes, and no evidence can support the existence of discrete subtypes^81^. Therefore, in some cases, the expression data of patients is potentially enriched in several pathways among different subtypes, showing a profile of mixed CMS signatures. When we performed the CRCSC classification of the TCGA dataset, we did not observe such a large proportion of CMS1/2/3 patients exhibiting CMS4 signatures (data not shown). Therefore, MMAS-1 patients may be exhibiting a profile of mixed CMS signatures. Furthermore, the distribution of CRCSC assignments is similar, but the death events in Taiwanese CRC tend to be different from TCGA dataset, raising the question about whether the profile of mixed CMS signatures of MMAS-1 patients is relevant to CRC deaths. However, more samples are needed to demonstrate the significance in the death distributions between TCGA and Taiwanese datasets stratified by CRCSC or CRCA subtyping.

In the present study, we discovered that MMAS discriminates samples by the activity of WNT signaling and stratifies samples based on distinct outcomes. Furthermore, MMAS highlights the importance of the WNT signaling pathway underlying the miRNA–mRNA regulation axis to the clinical outcome. Several studies have reported that activated WNT signaling is correlated with poor outcome of CRC^82–85^. Surprisingly, we observed a poor prognosis associated with a suppressed WNT signaling. Melo *et al*. suggested that WNT target genes are silenced by methylation and exhibit a poor prognosis in CRC, which is partially in line with our observations^86^. In their study, they examined the prognosis in relapse-free survival from CRC and reported that repressed WNT signaling is associated with metastasis. In the present study, the poor prognosis is observed specifically in the OS, but not the DFS (Fig. 4E–F), and the number of stage 4 patients (with metastasis) tends to be higher in MMAS-1 (Fig. 5D, P value> 0.05). In support of this notion, Kim *et al*. also suggested that methylation of the WNT signaling target gene can predict poor prognosis of CRC.^87^. Collectively, this information strengthens the correlation between repressed WNT signaling and poor prognosis and further raises the possibility that the regulation of the miRNA–mRNA network may be associated or act coordinately with methylation.

By applying various filters, such as parameters of fold change, low abundance, and input size, we generated hundreds of combinations for the NMF clustering input for both mRNA and miRNA datasets. Then, we screened for the following: (1) sample groupings exhibiting significant differences in survival outcome from both datasets and (2) concordant sample grouping of both RNA molecule data. The rationale behind our approach is the use of either miRNA or RNA data to stratify samples, followed by the testing of the association between two grouping results to explore the clinically associated networks or connections between two regulatory layers. During the screening step, we found that if sample groupings show difference in terms of survival outcome, then the sample groupings between two datasets may show association in some cases, suggesting that the composition of the input contains not only the information of survival discrimination but also the information that connects two regulatory layers. Furthermore, we did not observe any sample grouping showing significant differences in DFS (data not shown). Events of DFS include death, recurrence, and local/distant metastasis, each of which may express distinct molecular pathways, and therefore, different sets of key signature genes. Due to this molecular heterogeneity, a simple clustering approach may not be sufficient in deconvoluting several clinical events all at once. Therefore, focusing on one clinical event at a time when performing similar analyses will result in improved resolution or accuracy.

## Conclusions

In the past decade, several transcriptomic CRC studies have been reported. Most of their outcomes, such as TCGA, are relevant to people of European ancestry, and little is known about the transcriptomic expression underlying the tumorigenesis of CRC in other ethnicities. In the present study, we collected CRC samples from Taiwanese patients and extended the knowledge of CRC through the following findings: (1) We elucidated the spectrum of CRC-associated dysregulated mRNA molecules and pathways. By integrating this with the miRNA dataset, we built an miRNA–mRNA network and identified the critical subnetworks potentially involved in CRC tumorigenesis. (2) By comparing with the TCGA dataset, we discovered differential enrichment of metastasis-associated pathways between datasets, despite moderate extent of correlated expression in gene patterns. (3) We identified and validated several fusion genes in our CRC cohort, including recurrent and novel events. (4) The subtype profile of Taiwanese CRC patients was revealed using the CRCSC and CRCA classifier tools. The resulting distribution of subtype assignments is similar to that from the TCGA dataset but shows distinct clinical outcomes. (5) We then proposed a new subtype classification approach, MMAS, which discriminates survival outcomes of our patients and links the repressed activity of the WNT signaling pathway. Our findings support our hypothesis of an ethnic basis for CRC tumorigenesis and, hence, provide prospects for translational medicine. Furthermore, to the best of our knowledge, this dataset provides the most comprehensive and integrated set of altered transcriptomic signatures in patients of Han-Chinese ethnicity.

## Methods

### Colorectal cancer samples and RNA extraction

A total of 104 matched tissues were obtained from colon adenocarcinoma/adenoma patients during surgery at Chang Gung Memorial Hospital, Linkuo, Taiwan. The tumor and adjacent normal tissues were preserved in RNAlater immediately after resection and stored at 4 °C. About 50~60 mg of tissues were used for RNA extraction using TRIzol Reagent according to the manufacturer’s instructions. The quality of RNA samples was evaluated by LabChip GX, and samples with scores of greater than 7 were further processed. The period of samples collection was from December of 2014 to June of 2016.

### Next-generation sequencing and data pre-processing

For RNA-seq, we prepared the libraries by Agilent SureSelect Strand-Specific RNA Library Preparation Kit for Illumina (Agilent Technologies) according to the manufacturer’s instructions. Briefly, 2 μg purified total RNA was enriched by the ploy-A tail beads, fragmentized by heat treatment and then reverse transcribed to cDNA using dUTP approach. Adapters ligated libraries were then PCR amplified and purified. For the small RNA-seq, we prepared libraries using Illumina TruSeq small RNA library preparation kits according to manufacturer’s instructions. Briefly, 1 μg of purified total RNA was ligated with adapter and PCR amplified. The small RNA libraries were size selected with a target insert size of 15~30 nt in length using the 6% TBE PAGE gel. The yield and size distribution of the purified RNA and small RNA libraries were assessed using the Agilent 2100 Bioanalyzer instrument with the Agilent High Sensitivity DNA Kit (Agilent Technologies). Equal amounts of libraries were pooled in molecular ratio and consequently sequenced by the NextSeq. 500 sequencer.

Adapter sequences from both small RNA-Seq and RNA-Seq experiments were trimmed by Skewer^88^. For small RNA-Seq, trimmed reads were mapped to hg38 reference by using Bowtie^89^ with the parameter of “-n 1 -l 15 -k 1”. miRNA counts were calculated using featureCounts 1.5.1^90^ with miRBase 21 with 10 bp padding. For RNA-Seq, we mapped RNA reads by using BaseSpace app: RNA-Seq Alignment v1.0 with STAR and hg38 reference. Aligned RNA-seq reads were quantified and annotated using featureCounts 1.5.1^90^ with GENCODE release 25. In addition, aligned RNA-Seq reads were analyzed using BAM-matcher to ensure that samples were properly paired^46^. Both RNA-Seq and small RNA-Seq raw read counts were normalized to reads per million (RPM) for quantitative representation. Principal component analysis (PCA) analysis was performed on both sequencing data sets using R package ‘ggbiplot’ and ‘stats’.

### Differential expression analysis

R/BioConductor ‘DESeq. 2’ was used to perform the differential expression analysis. We defined differentially expressed genes (DEGs) and miRNAs (DEMs) in the tumor/normal comparison on the basis of adjusted P value <0.001 and |fold change | >2. Because the fold changes of low abundance genes may not be unequivocally determined, we excluded genes with per kilobase per million mapped reads (RPKM) values of less than 10. For the comparison among MMAS groups, differential expression analysis was performed for the tumor samples and DEGs were defined by adjusted P value <0.05 and |fold change | >1.5. For the TCGA dataset, we downloaded all the colon adenocarcinoma (COAD) and rectum adenocarcinoma (READ) controlled-access RNA-Seq bam files from Genomic Data Commons (GDC). The quantification of read counts and differential expression analysis were performed as above.

### Pathway analysis

To explore cellular pathways perturbed between tumor and normal samples, we used R/BioConductor packages ‘clusterProfiler’ and ‘fgsea’ for the overrepresentation enrichment and Gene Set Enrichment analysis (GSEA), respectively^91^. The gene set annotations of Kyoto Encyclopaedia of Genes and Genomes (KEGG) was obtained from Molecular Signatures Database (MSigDB)^92^. The visualization of KEGG pathway was conducted by the R/BioConductor package ‘Pathview’. To investigate the dysregulated pathway for each patient sample, we used R package ‘CMScaller’ with curated CRC pathways and performed the single-sample Gene Set Enrichment analysis (ssGSEA) using R/BioConductor package ‘GSVA’^64,93^. The highly variable pathways were examined by median absolute deviation (MAD). The top 35 highly variable pathways were represented with heatmap, while the top 20 variable pathways were subjected to PCA to inspect sample grouping by using R package ‘ggbiplot’ and ‘stats’.

### Gene fusion prediction and validation

We used STAR-Fusion^58^ 1.3.1 to uncover potential gene fusion events based on the Genome Resource Lib of GRCh38_v27_CTAT_lib_Feb092018. We executed the STAR-Fusion on the fastq files with the parameters of “–FusionInspector validate” and “–examine_coding_effect” to obtain *in silico* predicted fusion transcript sequences. We further performed Pfam^94^ to extend the functional domains annotations. For identification criteria, we filtered for fusion genes that are (1) in-frame transcripts, (2) with junction reads count> 5, and (3) without analogous fusion detection in the matched normal sample. For a high-confidence fusion gene set, we further annotated fusions that were observed in either CCEL, Cosmic, FA_CancerSupp, Chimer, YOSHIHARA_TCGA and Klijn_CellLines (STARFusion built-in databases). For validation, we adopted RT-PCR and Sanger sequencing approaches. One μg of total RNA was reverse transcribed to cDNA using SuperScript III Reverse Transcriptase kit (Invitrogen) according to the manufacturer’s instructions. Primers designed to detect specific fusions were as follows: ERBB2-PPP1R1B-F: AGA CAC GTT TGA GTC CAT GC, ERBB2-PPP1R1B-R: CTG GCC ACA GGT TGT CTT T; FGFR3-TACC3-F: AGA GGC CCA CCT TCA AGC, FGFR3-TACC3-R: ACT GCC TGG ACA GCT TGT G; PTPRK-RSPO3-F: TCT TGC TCC TCT CTC CTT GG, PTPRK-RSPO3-R: CAT GTT GCA CAG CCT CCT T; RPS19-CEACAM5-F: GGT GGA TAC CGT CAA GCT G, RPS19-CEACAM5-R: CAG CTG AGA GAC CAG GAG AAG; TBC1D15-RAB21-F: AAG CAG AAT GGG ACA TGG TT, TBC1D15-RAB21-R: TGC ATA CGA CTC TGC TTC TTG; TPM3-NTRK1-F: GAG TTT GCT GAG AGA TCG GTA G and TPM3-NTRK1-R: GTG TTT CGT CCT TCT TCT CCA. cDNA was amplified by using KAPA HiFi PCR kit (KAPABIOSYSTEMS) (Figure S7), cloned into pHE Vector (BIOTOOLS) and subjected to Sanger sequencing.

### miRNA-mRNA network analysis

To perform miRNA-mRNA network analysis, we first built a miRNA-target interactions (MTIs) database by collecting miRNA-mRNA pairs annotated by both TargetScan 7.1 and miRTarBase 7.0 as the basis for confident miRNA-target prediction^49,50^. A total of 25,892 MTIs was curated by this database. For the miRNA-mRNA network analysis between normal and tumor tissues, we calculated Pearson’s correlation and considered inversely correlated interactions (*r* < 0, P value <0.05) as putative targets for downstream analyses. For the MMAS miRNA-mRNA network analysis, the same analysis pipeline was used on MMAS-1 and MMAS-2 assigned tumor samples. The visualization of constructed miRNA-mRNA network was conducted by the R package ‘visNetwork’. The betweenness scores were calculated by the R package ‘igraph’.

### CRC transcriptome subtype analysis

To perform CRCSC classification, we used the R package ‘CMScaller’ implemented with the nearest template prediction algorithm (NTP) and the built-in classifier^64^. To generate the CRC*-*assigner (CRCA) classifier, the CRCassigner-786 gene list was obtained from the report by Sadanandam *et al*.^37^. In addition to the publicly available tools, we also intended to categorize patient subtypes based on our own transcriptome sequencing data, which included both mRNA and miRNA information. To this end, we utilized the non-negative matrix factorization (NMF) for subtype identification. In addition to the conventional NMF clustering approach, which uses highly variable genes as the input, we applied several filters to obtain the screening input genes/miRNA set: input size, fold change cutoff, and low abundance cutoff. For the input size filter, we calculated MAD for the mRNA and miRNA datasets. We then generated mRNA input sets with sizes ranging from 100 to 1500 of the most variable mRNAs based on MAD (with increment of 100). Similar criteria were applied for the miRNA input size, but the size intervals were changed to 25 ~ 600 with increment of 25. For the fold change cutoff, we used |fold change | >1.5 or 2 in the tumor versus normal comparison (with FDR < 0.05) or nil criterion. For the low abundance cutoff, we retained mRNAs/miRNAs with zero counts in <25%, 50%, 75%, or 100% of cohort size. Consequently, these filters generated 180 and 288 combinations for mRNA and miRNA inputs, respectively.

We then performed NMF clustering (by k-mer = 2, Brunet method)^95^ based on these screening input sets, and applied the following two criteria: (1) survival analysis of the stratified groups showing significant differences (log-rank test, P value < 0.05); (2) significant association between sample groupings based on the mRNA and miRNA sets (Fisher’s exact test, P value < 0.05). For effective use of the computing resources, we first ran the NMF with nrun = 25 to screen for the overall or disease-free survival (OS or DFS; P value <0.075). Subsequently, initially passed input sets were subjected to NMF again with nrun = 500 to screen for input sets that meet the criteria described above. We chose the input set by the most significant log-rank test results. Consequently, the attributes of the mRNA input set were: gene size of 400, |fold change | >1.5 and genes with zero counts less than 25% of sample size, whereas the miRNA input is characterized by size of 50, |fold change | >2 and genes with zero counts in less than 25% of samples.

### Reconstruction of transcriptional network analysis

In order to identify risk regulons associated with MMAS, we used R/BioConductor package ‘RTN’ to identify putative transcription factor (TF) regulons. Subsequently, R/BioConductor package ‘RTNsurvival’ was conducted to compute the individual regulon activity, which was further stratified for the survival analysis. Any association between MMAS and regulon activity was examined by using Fisher’s exact test.

### Statistical analysis

To test the association between clinical attributes, such as sex, TNM stage, location, CEA, alcohol, family cancer history, histological grade and smoke, and presence of fusion or MMAS, Fisher’s exact test was used and P value < 0.05 was considered as a significant association. For patient survival analyses (both OS and DFS), Kaplan-Meier method with log-rank test and Cox proportional hazard models were performed by using the R package ‘survival’ and ‘survminer’. Stage-four patients were not included for the DFS analysis.

### Ethics approval and consent to participate

This study was approved by the Chang Gung Memorial Hospital Institutional Review Board as a retrospective analysis (IRB 103-2529B) and was conducted within the guidelines of the Declaration of Helsinki. Patients/families were counseled in the context of the present study design, and all participants provided written informed consent to participate in the study.

### Consent for publication

All individuals involved in this study provided consent for publication. We also obtained consent to publish the clinical information of all individuals presented in this study.

## Supplementary information

Supplementary Information.

Supplementary Tables.

## Data Availability

The fastq format data of RNA-seq and small RNA-seq were deposited at NCBI Sequence Read Archive (SRA) database with the project accession number PRJNA387172.
