# Supplementary material for: Comprehensive transcriptome profiling of Taiwanese colorectal cancer implicates an ethnic basis for pathogenesis
Source: Sci Rep. 2020 Mar 11;10:4526. doi: 10.1038/s41598-020-61273-y (PMC7066141; doi:10.1038/s41598-020-61273-y)
Supplement: Supplementary file 1 — Supplementary Information. [file 41598_2020_61273_MOESM1_ESM.doc]

**Supplementary Information for:**

**Comprehensive transcriptome profiling of Taiwanese colorectal cancer implicates an ethnic basis for pathogenesis**

Shao-Min Wu^1^, Wen-Sy Tsai^2^, Sum-Fu Chiang^2,3^, Yi-Hsuan Lai^1,4^, Chung-Pei Ma^1,4^, Jian-Hua Wang^1^, Jiarong Lin^5^, Pei-Shan Lu^5^, Chia-Yu Yang^1,5,6,7^, Bertrand Chin-Ming Tan^1,4,8,9^, and Hsuan Liu^1,2,5,10^

^1^Graduate Institute of Biomedical Sciences, College of Medicine, Chang Gung University, Taoyuan, Taiwan

^2^Division of Colon and Rectal Surgery, Lin-Kou Medical Center, Chang Gung Memorial Hospital, Taoyuan, Taiwan

^3^Graduate Institute of Clinical Medical Sciences, College of Medicine, Chang Gung University, Taoyuan, Taiwan

^4^Department of Biomedical Sciences, College of Medicine, Chang Gung University, Taoyuan, Taiwan

^5^Molecular Medicine Research Center, Chang Gung University, Taoyuan, Taiwan

^6^Department of Microbiology and Immunology, College of Medicine, Chang Gung University, Taoyuan, Taiwan

^7^Department of Otolaryngology-Head & Neck Surgery, Chang Gung Memorial Hospital, Linkou, Taoyuan, Taiwan

^8^Department of Neurosurgery, Linkou Medical Center, Chang Gung Memorial Hospital, Linkou, Taiwan

^9^Research Center for Emerging Viral Infections, Chang Gung University, Taoyuan, Taiwan

^10^Department of Cell and Molecular Biology, College of Medicine, Chang Gung University, Taoyuan, Taiwan


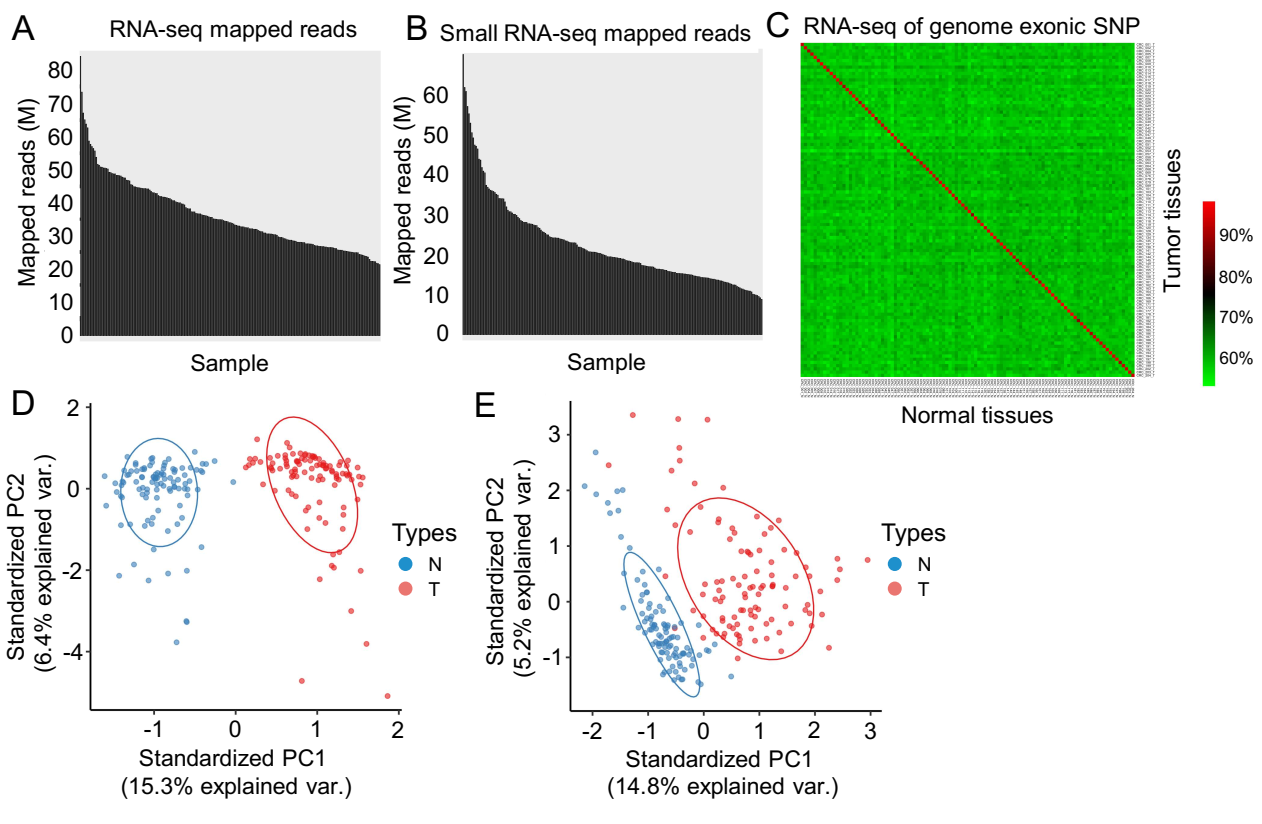


**Figure S1.** Sequencing data quality statistics.

Statistics of (A) RNA-seq and (B) small RNA-seq mapped reads. (C) Sample pairing quality control. RNA-seq bam files and the subset of 1000 genome exonic SNP with allele frequency 45%–55% were used for discriminating samples. The key color represents the fraction of common SNPs. Red and green represent high and low percentage of common SNP, respectively. Principal component analysis (PCA) of tumor and adjacent normal tissues for (D) RNA-seq and (E) small RNA-seq data. Red and blue dots indicate tumor and normal tissues, respectively.


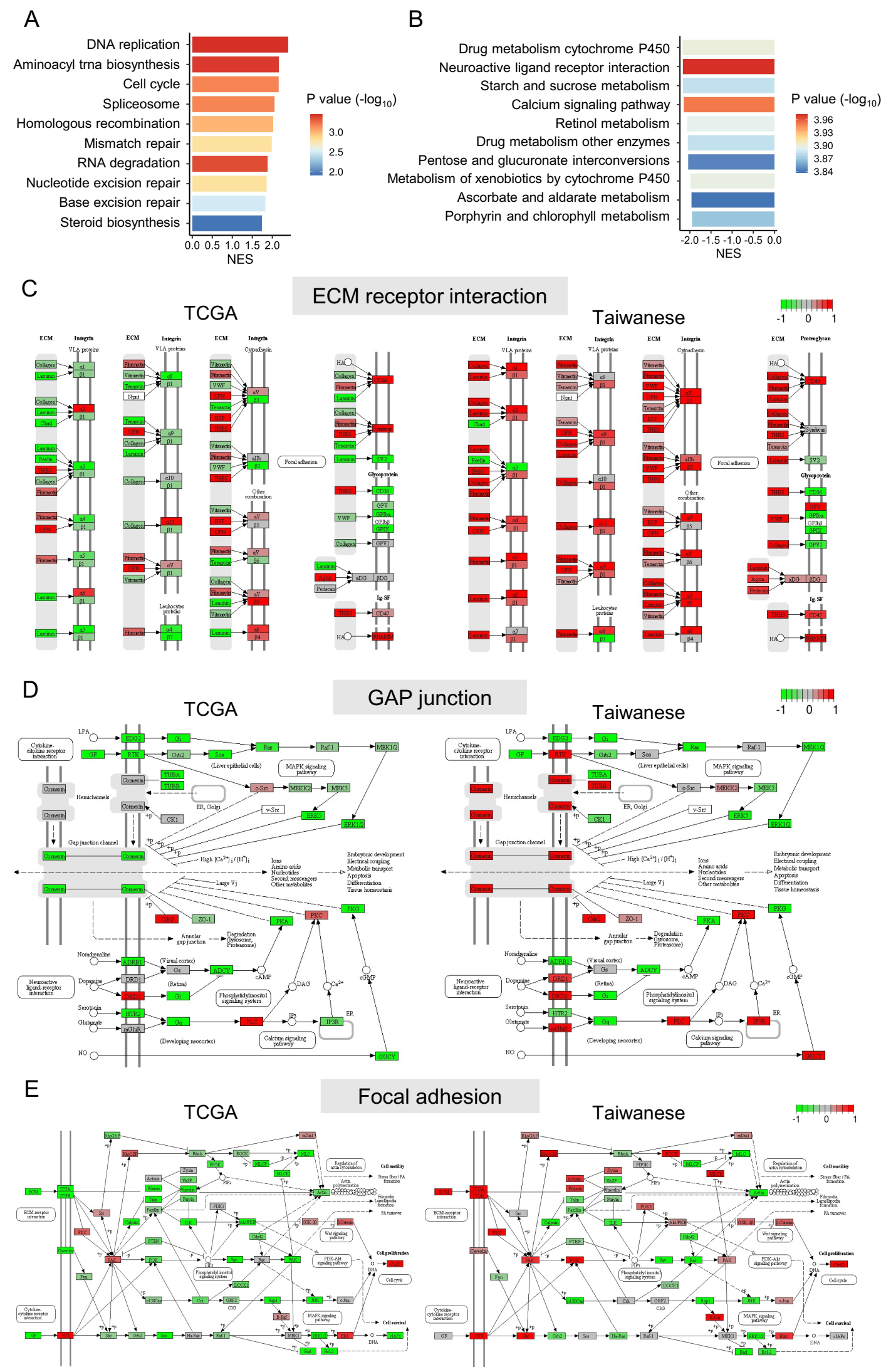


**Figure S2.** Comparison of TCGA and Taiwanese CRC dataset.

GSEA charts of top ten significantly enriched (P value < 0.05) upregulated (A, NES > 0) and downregulated (B, NES < 0) KEGG pathways. Visualization of distinct pathway expression patterns[^1-3^](#_ENREF_1) between TCGA (C, F, H) and Taiwanese (D, G, I) CRC datasets using Pathview for (C) ECM receptor interaction, (D) gap junction, and (E) focal adhesion pathways.


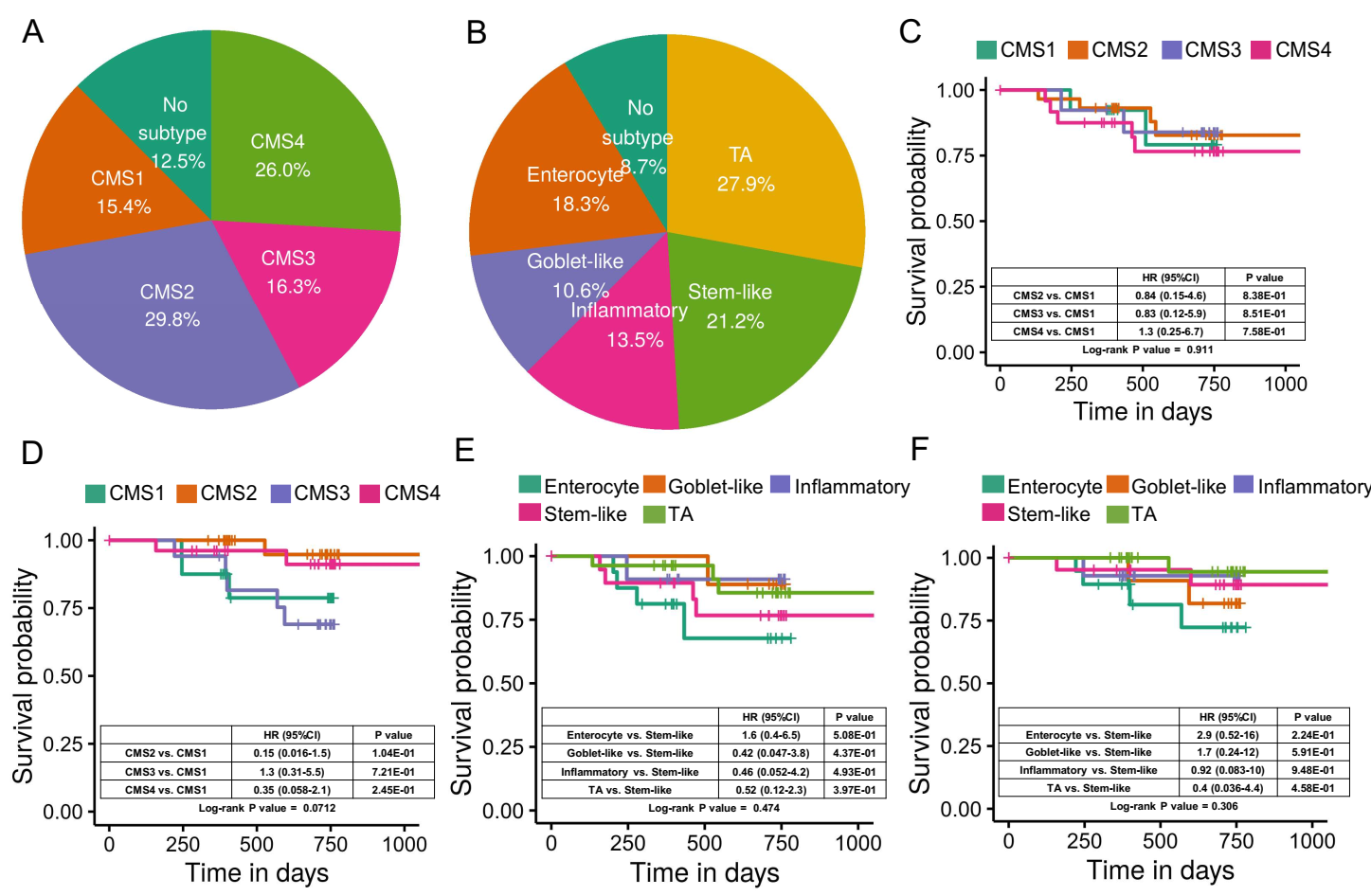


**Figure S3.** CRCSC and CRCA classification for Taiwanese CRC patients.

Distribution (n = 104) of (A) CRCSC and (B) CRCA classification for Taiwanese CRC patients. Kaplan–Meier plots of (C and E) DFS and (D and F) OS stratified by (C and D) CRCSC and (E and F) CRCA classification, respectively. The significance of the survival rate is compared using the log-rank test, and univariate analyses are performed to evaluate the hazard ratios (HR) and 95% confidence interval (CI) for each pairwise comparison.


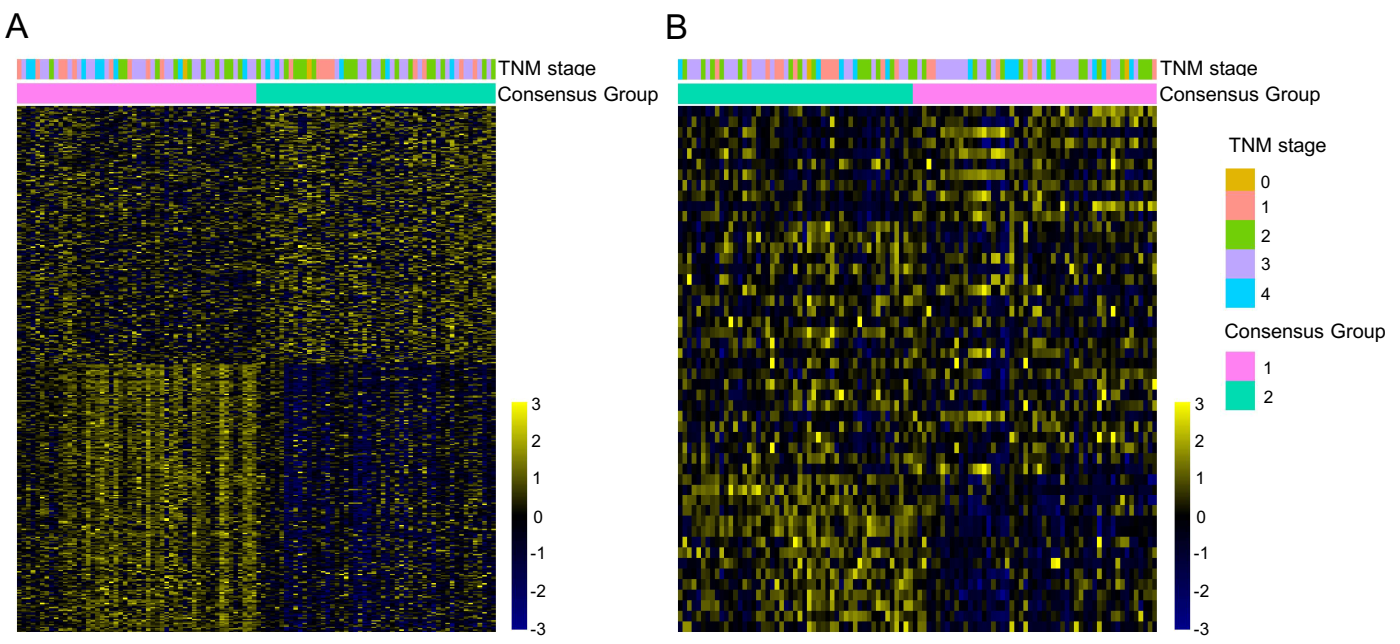


**Figure S4**. NMF clustering for Taiwanese CRC patients.

Heatmap of NMF clustering of (A) mRNA and (B) miRNA dataset for Taiwanese CRC patients.


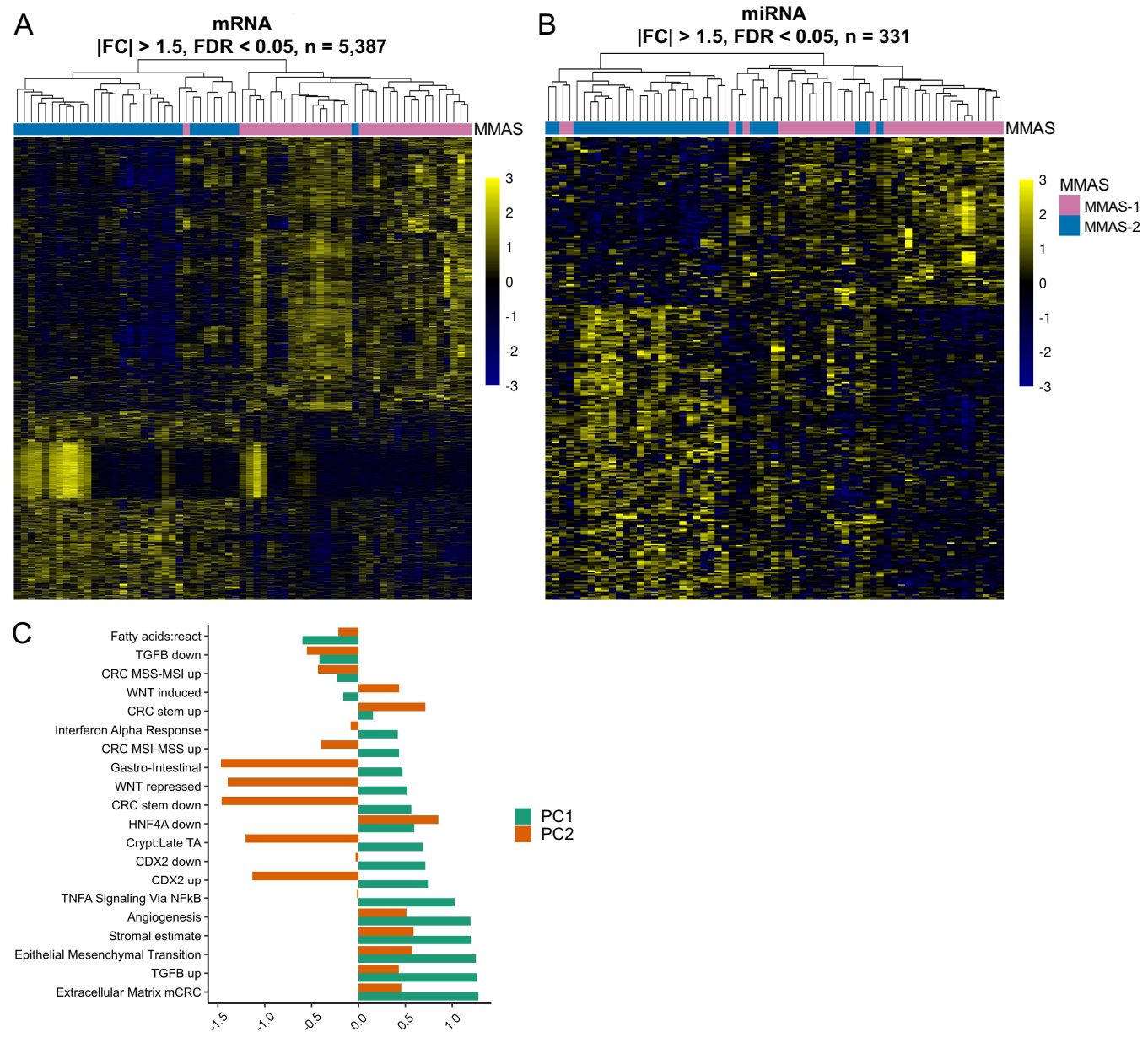


**Figure S5.** Distinct expression profiles between MMAS-1 and MMAS-2.

Hierarchical clustering of (A) DEGs and (B) DEMs between MMASs from tumor tissues of Taiwanese CRC patients (|fold change | >1.5, FDR < 0.05). (C) PC1 and PC2 loading bar plot of PCA derived from 104 tumor samples with top 20 most deviated pathways.


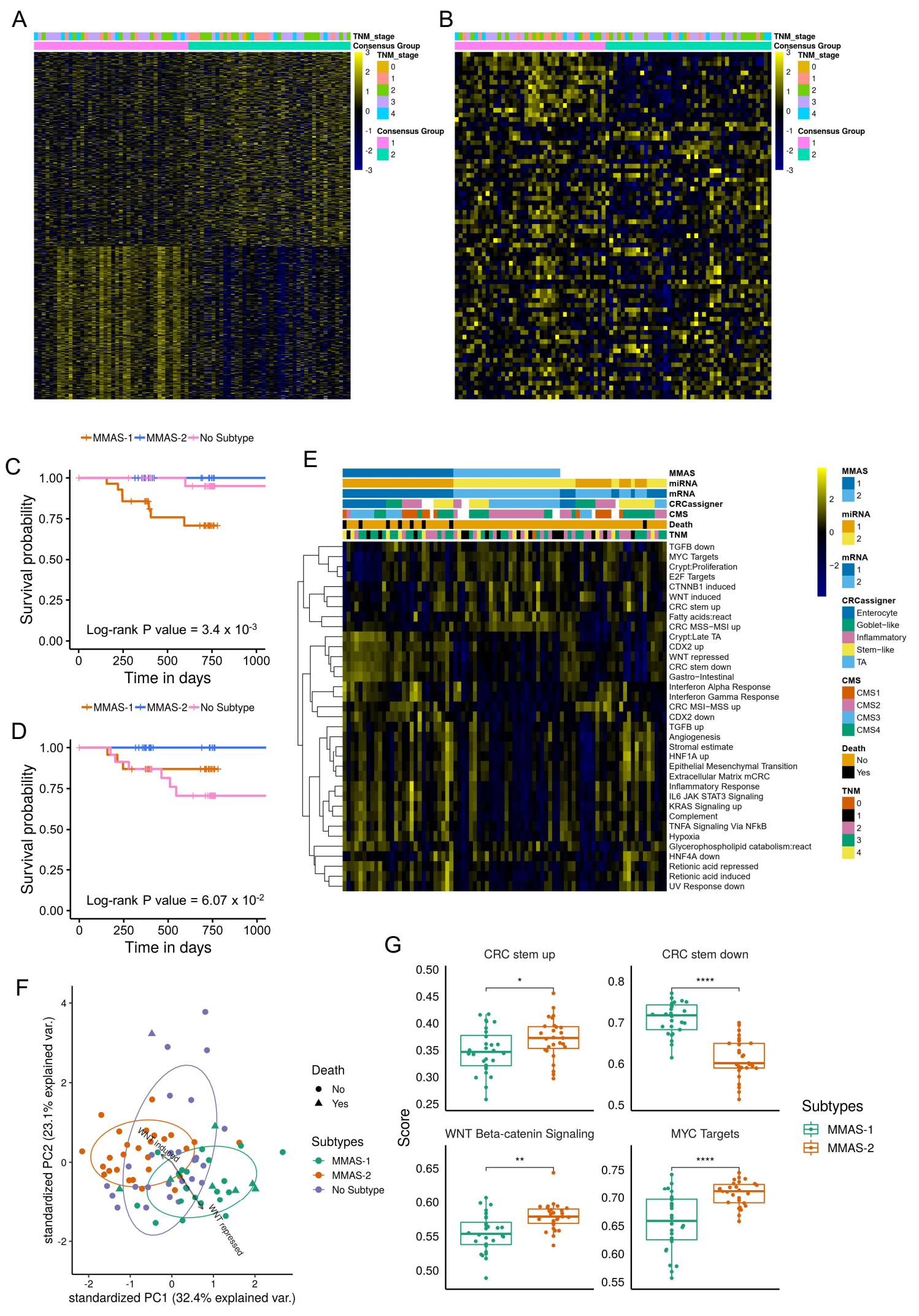


**Figure S6.** Evaluation of the MMAS approach by resampling.

Evaluation of MMAS data processing approach with resampling of 80% samples and event sampling ratio of 5 (OS). Analytic procedures were performed as described above (Figure S4A-B and Figure 5E-G). Heatmap of NMF clustering of (A) mRNA and (B) miRNA data for the resampled data set. (C) OS and (D) DFS analysis for resampled data set stratified by concordant assignment of mRNA and miRNA NMF clustering. (E) Heatmap of ssGSEA result for resampled data set with top 35 most deviated pathways is shown. (F) PCA of ssGSEA result for resampled data set. (G) Box plot of pathway enrichment score for CRC stem up, CRC stem down, WNT/β-catenin signaling, and MYC target pathways for resampled data set. (***, P value ≤ .001; ****, P value ≤ .0001, Mann–Whitney test.)

**Figure S7.** Uncropped gel images corresponding to the data shown in Figure 3B.

**References**

1 Kanehisa, M. Toward understanding the origin and evolution of cellular organisms. *Protein science : a publication of the Protein Society* **28**, 1947-1951, doi:10.1002/pro.3715 (2019).

2 Kanehisa, M. & Goto, S. KEGG: kyoto encyclopedia of genes and genomes. *Nucleic acids research* **28**, 27-30, doi:10.1093/nar/28.1.27 (2000).

3 Kanehisa, M., Sato, Y., Furumichi, M., Morishima, K. & Tanabe, M. New approach for understanding genome variations in KEGG. *Nucleic acids research* **47**, D590-D595, doi:10.1093/nar/gky962 (2019).
